# Dengue: The break-bone fever outbreak in Kerala, India

**DOI:** 10.3126/nje.v7i2.17972

**Published:** 2017-06-30

**Authors:** Indrajit Banerjee

**Affiliations:** 1 Associate Professor, Department of Pharmacology, Chitwan Medical College and Teaching Hospital Bharatpur, Nepal

## Global disease burden of Dengue

WHO has categorized Dengue as the quickest spreading arboreal disease in the world and about 50% the world ' s population is at jeopardy of getting affected by the deadly viral manifestations [ 1, 2, 3 ]. Dengue is a daunting global disease burden and economic burden throughout the globe [ 4 ]. Dengue is produced by one of 4 single stranded, positive sense RNA viruses, serotype DENV1-4 of family Flaviviridae and the genus flavivirus [ 5, 6 ]. The morphology of this RNA virus comprises of 3 structural proteins-capsid, envelope, glycoproteins and 7 nonstructural proteins NS1, NS2A, NS2B, NS3, NS4A, NS4B and NS5 [ 7 ]. Dengue is primarily transmitted by the mosquito Aedes aegypti but in recent year ' s infection with Aedes albopictus has intensely increased [ 8 ]. Dengue has two clinical manifestations, dengue hemorrhagic fever and dengue shock syndrome. Nowadays it is known as severe dengue [ 9 ]. According to Stanaway JD et al. a total number of 60 million symptomatic dengue infections were present around the globe and resulting in about 10 000 deaths in the year 2013 [ 10 ]. This figure has been reduced as compared to the Global Burden of Disease Study, which has assessed that in 2010, 14 000 people has died from dengue [ 11 ]. Whereas according to WHO, Dengue is predominant in 128 countries and 4 billion people were at danger across the globe. Dengue affects 50 million to 100 million people per year and subsequently 20 000 deaths per year [ 12 ]. It is estimated that in Southeast Asia there are about 21-1 million cases of Dengue. [ 7 ] Dengue and dengue hemorrhagic fever is commonly found in Pacific, Caribbean islands, North America, South America and in Southeast Asia [ 13 ].

## Dengue in Kerala

Recently a shocking intensification of dengue cases has also been seen in India in 2017 [ 14 ]. The common serotype of Dengue Asian genotype of DENV-1 was detected in South India in 2012 [ 15 ]. In Orisha all four serotype of the virus has been detected and reported in 2017 by Mishra B et al [ 16 ]. In the year 2017, approximately 18,700 cases of Dengue have been confirmed from India which was quiet alarming. Kerala was at the top of the list which was having the highest number reported from India. The total number of Dengue patients in Kerala was 9104 followed by Tamil Nadu 4174, Karnataka 1945 cases, Gujarat 616, Andhra Pradesh 606, West Bengal 469, Delhi 100 till 2nd July 2017 [ 17 ]. According to the Times of India, the daily newspaper the most number of cases in the state Kerala was reported from the districts of Thiruvananthapuram, Kozhikode and Palakkad. The possible reason was due to drought in South India and people stored drinking water in large containers to overcome the water crisis and stagnant rainwater have led to an outburst in mosquito growth in the state with a total death toll reaching around 115 in the year 2017 [ 18, 19, 20 ].

## Clinical Features, Investigations and findings of Dengue

The common dengue symptoms are sudden high grade fever 103-106° F (Continuous or Saddle back temperature curve), headache, joint pain, myalgia, retro orbital pain, maculopapular rashes starts from the chest and extends to the extremities sparing palms and the soles, involves the face later in the febrile stage. Mild hemorrhagic manifestations viz. petechiae, bleeding gums, epistaxis, menorrhagia, hematuria. Later, bleeding from gastrointestinal tract and sites of trauma, thrombocytopenia, pleural effusion, abdominal pain, restlessness, vomiting and a sudden reduction in temperature, adynamia and fainting can occur [ 7, 21 ]. Leukopenia, thrombocytopenia, elevation of serum aminotransferase level are seen. Detection of IgM, IgG antibodies, NS1 ELISA or duplex Real time polymerase chain reaction during the acute phase are used as diagnostic tests. Virus can be isolated from the blood by mosquito inoculation or mosquito cell culture [ 21, 22, 23 ].

## Treatment of Dengue

Dengue is a self-limiting viral infection. There is no specific antiviral drug currently available in the market for the treatment of Dengue. Analgesics, fluid replacement with Ringer ' s lactate, and bed rest are usually satisfactory [ 24 ]. Paracetamol is used as antipyretic in the patients. Aspirin is contraindicated as it may aggravate the chances of bleeding. Breeding places of Aedes mosquitoes should be abolished and the adults should be destroyed by suitable use of insecticides [ 25 ].

## Vaccination against Dengue

Dengvaxia (CYD-TDV) a tetravalent, recombinant, live, attenuated vaccine. This dengue vaccine is approved in Mexico, Philippines, Brazil, El Salvador and Paraguay [ 26, 27 ]. The vaccine was found to be efficacious in five Latin American countries in VCD and severe VCD cases. This vaccine is given in three dose schedule of 0, 6 and 12 months can be used in between 9-45 years of age. In India Dengvaxia is still not used as it has to undergo Phase III clinical trial [ 27 ]. 

 The disease burden of Dengue can be decreased to some extent by correct vaccination to the high risk groups, adequate mosquito control, early detection and appropriate treatment of Dengue cases in India in general and particularly in Kerala in future.

## Abbreviations

ELISA- enzyme-linked immunosorbent assay 

 VCD- Virologically confirmed dengue 

 WHO- World health Organization
